# The role of tDCS in psychiatrists toolbox

**DOI:** 10.1192/j.eurpsy.2023.165

**Published:** 2023-07-19

**Authors:** K. Järventausta

**Affiliations:** Clinical Medicine, Tampere University, Tampere, Finland

## Abstract

**Abstract:**

tDCS is a low-cost and well-tolerated neuromodulation treatment of depression. It seems to be a good and effective option to start the treatment of depression along or instead of medication in mild or moderate depression. tDCS can be used as an add-on treatment with psychotherapy or medication. Home based tDCS is easy to conduct. However, tDCS should be used with qualified protocols like any other neuromodulation treatment. When the tools in psychiatry are diversifying the precise diagnostics, careful defining of the clinical picture and effective early and individually planned management of depression are key elements gaining better outcomes and preventing treatment resistant depression.

**Disclosure of Interest:**

None Declared

